# A National Case-Crossover Study on the Risk of Kidney Injury Requiring Dialysis after Sepsis

**DOI:** 10.3390/jcm12154950

**Published:** 2023-07-27

**Authors:** Chung-Shun Wong, Tzu-Ting Chen, Andrei R. Akhmetzhanov, Ping-Jen Hu, Mai-Szu Wu, Mei-Yi Wu

**Affiliations:** 1 Department of Emergency Medicine, Shuang Ho Hospital, Taipei Medical University, New Taipei City 23561, Taiwan; 2 Department of Emergency Medicine, School of Medicine, College of Medicine, Taipei Medical University, Taipei 110, Taiwan; 3 Center for Neuropsychiatric Research, National Health Research Institutes, Miaoli County 35053, Taiwan; 4 Institute of Epidemiology and Preventive Medicine, College of Public Health, National Taiwan University, Taipei 106, Taiwan; 5 Global Health Program, College of Public Health, National Taiwan University, Taipei 106, Taiwan; 6 Division of Gastroenterology, Department of Internal Medicine, Shuang Ho Hospital, Taipei Medical University, New Taipei City 23561, Taiwan; 7 Division of Nephrology, Department of Internal Medicine, Shuang Ho Hospital, Taipei Medical University, New Taipei City 23561, Taiwan; 8 Division of Nephrology, Department of Internal Medicine, School of Medicine, College of Medicine, Taipei Medical University, Taipei 110, Taiwan; 9 TMU Research Center of Urology and Kidney, Taipei Medical University, Taipei 110, Taiwan

**Keywords:** sepsis, acute kidney injury, chronic kidney disease, acute temporary dialysis, chronic dialysis

## Abstract

Background: Patients with sepsis-associated acute kidney injury (AKI) are at risk of kidney damage, potentially necessitating acute temporary or chronic dialysis. Our study aims to estimate the odds ratio (OR) of preceding sepsis among patients requiring their first dialysis. Methods: A nationwide population-based case-only study was conducted using claims records from the National Health insurance database of Taiwan. All patients over 20 years of age who underwent their first dialysis between 2004 and 2016 were included in the study. The six months prior to their first dialysis served as a self-control period. Results: The study included 147,201 patients who required acute temporary and 75,031 patients who required chronic dialysis. The odds ratios for patients needing acute temporary dialysis after 1, 2, 3, and 4 weeks of exposure periods were 15.8, 10.7, 9.2, and 8.4, respectively. The ORs for patients requiring chronic dialysis were 7.0, 4.1, 4.2, and 3.7, respectively. Conclusions: Our findings indicate that sepsis was substantially associated with an increased risk of renal failure. The risk was highest during the first week following sepsis for both acute temporary and chronic dialysis cases.

## 1. Introduction

Sepsis, a life-threatening organ dysfunction, is a dysregulated host response to infection that can progress to septic shock, leading to multiple organ failure and high mortality rate of 54% [1]. Acute kidney injury (AKI) is a serious complication of sepsis, affecting more than 50% of cases [2]. In patients with sepsis and septic shock, AKI has a negative impact on prognosis and is associated with high mortality rates, prolonged stays in the intensive care unit and hospital, an increased risk of progressive chronic kidney disease (CKD), and a higher risk of death [3,4,5].

The incidence of AKI is positively correlated with the severity of sepsis [6,7]. A cohort study conducted in Spain showed a significant increase in AKI incidence depending on the severity of sepsis (4.2% for sepsis, 22.7% for severe sepsis, and 52.8% for septic shock) [6]. Another prospective cohort study including 1177 patients from 198 intensive care units in 24 European countries reported that up to 51% of patients with sepsis developed AKI [7]. AKI severity is determined based on changes in serum creatinine levels and urine output [8]. Patients with severe kidney injury may require dialysis to improve survival and outcomes. Although the association between sepsis and AKI is well established [9,10], research on the risk of kidney injury necessitating dialysis in patients with sepsis remains limited.

Chronic kidney disease (CKD) is a public health concern due to its rising incidence and prevalence, resulting in poor outcomes and substantial costs [11,12]. Patients with CKD are at an increased risk of sepsis-associated complications, including AKI [13,14]. Some studies have suggested that the progression of CKD may be accelerated by sepsis and sepsis-associated AKI [15,16].

We conducted a nationwide case-only study based on within-person comparisons by using a large national cohort in Taiwan. Our analysis employed claims data from the National Health Insurance (NHI) database, which has proven suitable for small- to large-scale studies, including assessment of global burden of diseases [17,18,19]. Our study included all patients over the age of 20 who underwent their first dialysis between 2004 and 2016 in Taiwan. We implemented a case-crossover study design using a six-month period prior to the first dialysis as a self-controlled comparison. This design allowed for implicit adjustment of confounding factors that do not change significantly over time, such as body mass index, smoking history, and dietary habits. Our objective was to investigate the risk of acute temporary and chronic dialysis following sepsis and to evaluate the effect of sepsis on acute dialysis in patients stratified by CKD.

## 2. Materials and Methods

Data sources

Data were retrieved from the National Health Insurance (NHI) database in Taiwan. Established in 1995, the NHI program covers 99.9% of the Taiwanese population of 23 million people [20,21]. The database contains comprehensive patient information, including date of birth, sex, residential or work area, dates of clinical visits, and corresponding diagnosis codes assigned according to the *International Classification of Diseases, Ninth Revision, Clinical Modification* (ICD-9-CM). It also includes prescription details, expenditure amounts, and outcomes at hospital discharge (recovered, died, or transferred). The study was approved after full review by the Joint Institutional Review Board of Taipei Medical University (TMU-JIRB N201909046) and was conducted in accordance with approved guidelines. Informed consent of the study participants was not required because all data were anonymized prior to use.

Study design and participants

**Figure 1:**
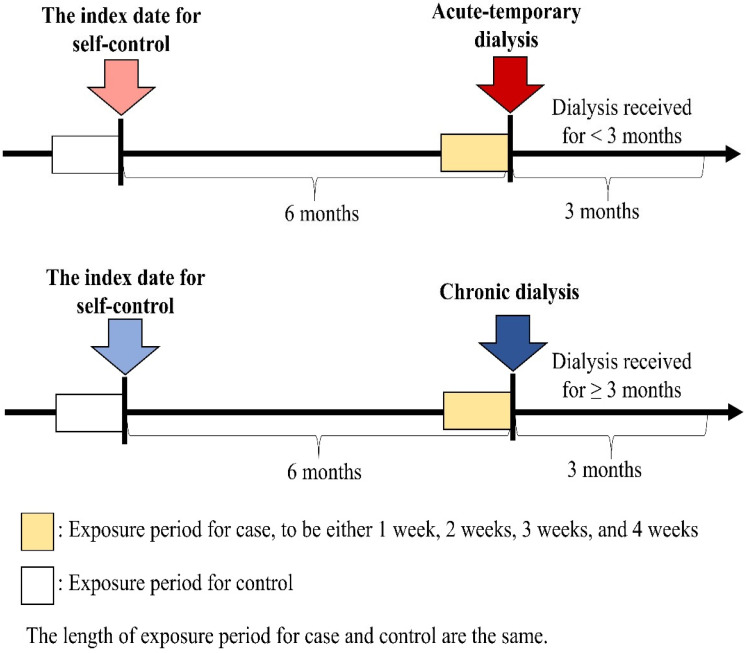
Timeline for case-crossover study design.

This national case-crossover study [22,23] analyzed data from all individuals who underwent their first dialysis between 1 January 2004 and 31 December 2016. Cases younger than 20 years old or with missing information on age or sex were excluded from the study. The primary outcome of interest was identified by the procedure codes for charges related to dialysis during hospital admission. Dialysis patients (cases) were then categorized into two types: acute temporary (dialysis received for less than three months) or chronic (dialysis received for more than three months) [24]. The selected cases constituted both the case group and the control group, as illustrated in Figure 1. The index date for the case group was the date of their first dialysis, while the index date for the control group was six months prior to the first episode. The exposure periods for both case and control groups were set to be one, two, three, or four weeks long, immediately preceding the respective index date. If sepsis causes kidney injury necessitating dialysis, we may observe a higher frequency of exposure periods with sepsis events in the case group compared with the control group. Conversely, if the frequency of exposure periods with sepsis events for cases is similar to that for controls, then sepsis would be considered independent of the risk of dialysis. The sepsis events were defined according to the Surviving Sepsis Campaign guidelines 2004, which refers to a systemic inflammatory response syndrome (SIRS) caused by infection. Sepsis events were identified using ICD-9-CM codes, 038.xx, 995.91, 995.92, or 785.52 in the database for the emergency department or inpatient care records.

Measurement of covariates

Our study included the following covariates: major risk factors for dialysis, such as diabetes mellitus (ICD-9-CM code: 250), hypertension (ICD-9-CM codes: 401–405), hyperlipidemia (ICD-9-CM codes: 272.0–272.4), and Charlson comorbidity index (CCI) [25,26]. Comorbidities were identified using at least one inpatient diagnosis with ICD-9-CM codes or three or more outpatient diagnoses with ICD-9-CM codes within one year before the index date. Concomitant medication use within one year prior to the index date was considered as a time-varying confounder, including nonsteroidal anti-inflammatory drugs (NSAIDs).

Subgroup and sensitivity analysis

We conducted subgroup analyses based on sex and the comorbid condition of CKD [27]. Patients with CKD (ICD-9-CM codes: 250.4, 274.1, 403.1, 404.2, 404.3, 440.1, 442.1, 447.3, 572.3, 642.1, 646.2, 572.4, 283.11, 580, 581, 582, 583, 584, 585, 586, 587, 588, 589, 403, and 404) were those who received a CKD diagnosis three times during visits to outpatient clinics or emergency departments or once during hospital admission. In sensitivity analysis, we evaluated the risk of different exposure period durations before the index date, including 1, 2, 3, and 4 weeks prior to the index date for both cases and controls. Additionally, we assessed the frequency of sepsis events at nonoverlapping time intervals before the index date, such as 1–7, 8–14, 15–21, and 22–28 days. Acute temporary and chronic dialysis were studied separately for each period.

Statistical analysis

A conditional logistic regression model was used to estimate the effect of sepsis on the risk of dialysis (odds ratios (ORs)) by conditioning on each sample. The baseline effect was cancelled out in both denominator and numerator because we utilized self-controls. As a result, the model controlled for the impact of baseline characteristics on the risk of dialysis, which is a known advantage of the case-crossover study design. According to the exposure status, samples can be divided into four groups: (Y, Y), (N, N), (Y, N), and (N, Y). The first entry indicates whether a sepsis event occurred in the case exposure period, and the second entry indicates whether a sepsis event occurred in the control exposure period. N indicates no, and Y indicates yes, namely a sepsis event occurred. (Y, Y) and (N, N) are concordant pairs, and (Y, N) and (N, Y) are discordant pairs. For example, we identified a sepsis event within the case exposure period but not within the control exposure period, and then we took this patient as a discordant pair in group (Y, N). Then, the odds ratio was derived from the number of (Y, N) pairs divided by the number of (N, Y) pairs, which was only based on the information of discordant pairs but not the concordant pairs. All analyses were performed using SAS Version 9.4 (SAS Institute Inc., Cary, NC, USA).

Data availability

The data that support the findings of this study are available from the National Health Insurance Research Database in Taiwan. Restrictions may apply to their availability, because the data were acquired under the license for the current study. Data cannot be shared by the authors or made public for ethical/privacy reasons.

## 3. Results

**Figure 2:**
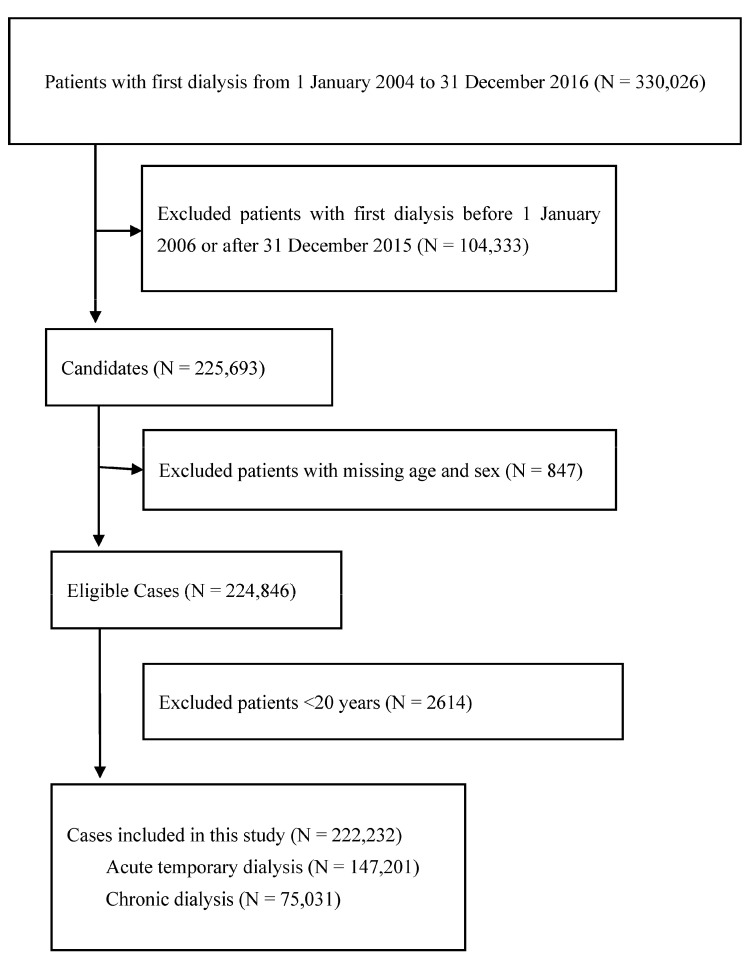
Flow diagram demonstrates the selection criteria and process of eligible dialysis patients.

Figure 2 presents the flow diagram for this case-crossover study. Patients with kidney injury who required their first dialysis between 1 January 2004 and 31 December 2016, were included in this study (N = 330,026). We excluded patients with their first dialysis before 1 January 2006 or after 31 December 2015 (N = 104,333), with missing age and sex data (N = 847), or those aged <20 years (N = 2614), resulting in 222,232 eligible patients. A total of 147,201 and 75,031 patients with kidney injury required acute temporary and chronic dialysis, respectively. Table 1 displays the characteristics of patients with kidney injury who required acute temporary and chronic dialysis.

The mean ages of patients with kidney injury requiring their first dialysis in the acute temporary and chronic dialysis groups were 68.2 ± 15.7 and 63.7 ± 14.5 years, respectively. The frequencies of comorbidities such as diabetes mellitus, hypertension, and hyperlipidemia were slightly higher in the case group compared with the control group. The demographic characteristics of patients receiving acute temporary dialysis stratified by CKD are shown in Table 2.

**Figure 3:**
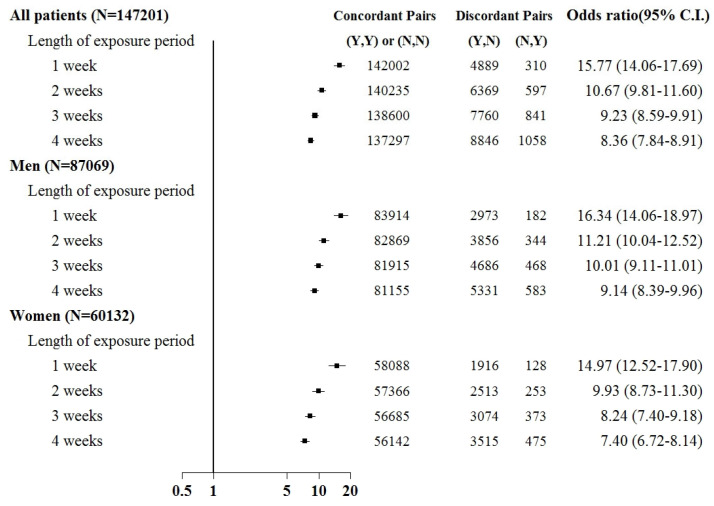
Odds ratios of acute temporary dialysis after sepsis when control was selected from six months before first dialysis. For the concordant and discordant pairs, the first and second entries indicate whether a sepsis event occurred in the case or control within an exposure period, respectively. N indicates no, and Y indicates yes, namely a sepsis event occurred.

**Figure 4:**
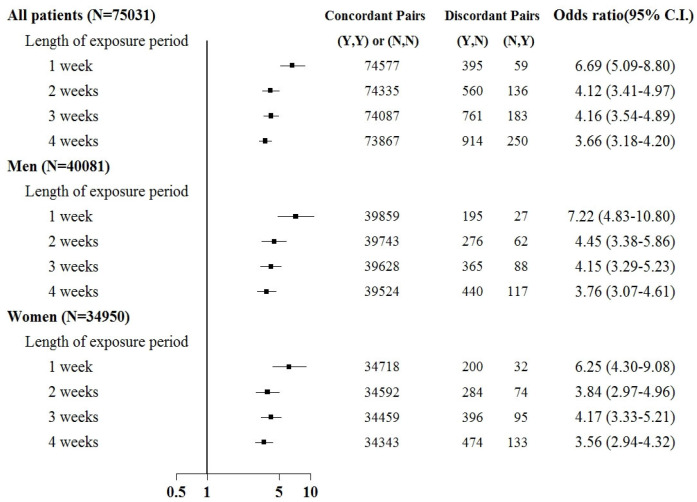
Odds ratios of chronic dialysis after sepsis when control was selected from six months before first dialysis. For the concordant and discordant pairs, the first and second entries indicate whether a sepsis event occurred in the case or control within an exposure period, respectively. N indicates no, and Y indicates yes, namely a sepsis event occurred.

Sepsis was associated with an increased risk of kidney injury requiring acute temporary dialysis, with odds ratios (ORs) of 15.8 (95% confidence interval [CI]: 14.1–17.7), 10.7 (9.8–11.6), 9.2 (8.6–9.9), and 8.4 (7.8–8.9) for exposure periods of 1, 2, 3, and 4 weeks, respectively, as shown in Figure 3. The risk of requiring acute temporary dialysis persisted but the risk magnitude decreased as the time interval extended. The risk of receiving acute temporary dialysis remained significantly high after extending the time interval to 4 weeks. A similar trend was observed in patients with renal failure requiring chronic dialysis, where odds ratios (ORs) were 7.0 (95% CI: 5.1–8.8), 4.1 (3.5–4.9), 4.2 (3.5–4.9), and 3.7 (3.2–4.2) for exposure periods of 1, 2, 3, and 4 weeks, respectively, as shown in Figure 4.

**Figure 5:**
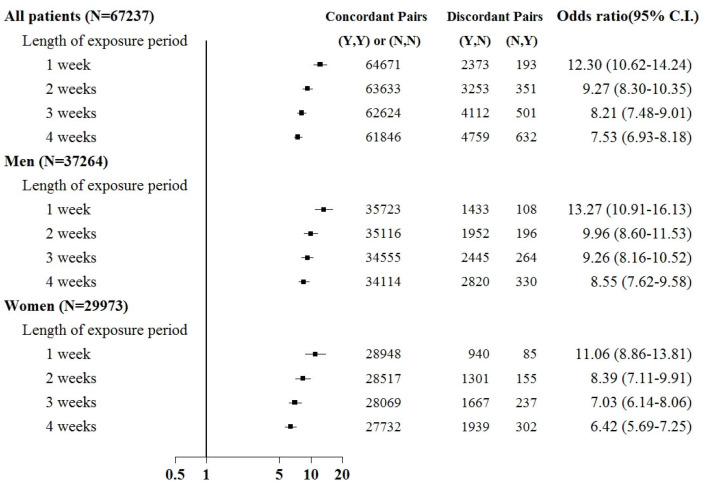
Odds ratios of acute temporary dialysis after sepsis in patients with chronic kidney disease when control was selected from six months before first dialysis. For the concordant and discordant pairs, the first and second entries indicate whether a sepsis event occurred in the case or control within an exposure period, respectively. N indicates no, and Y indicates yes, namely a sepsis event occurred.

**Figure 6:**
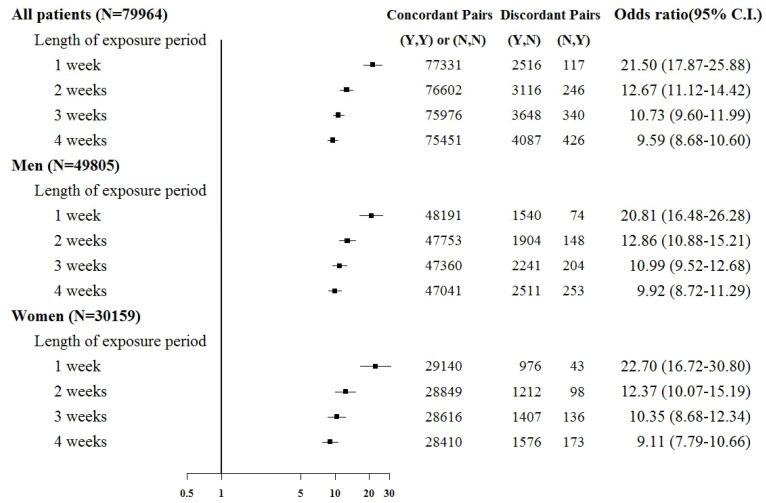
Odds ratios of acute temporary dialysis after sepsis in patients without chronic kidney disease when control was selected from six months before first dialysis. For the concordant and discordant pairs, the first and second entries indicate whether a sepsis event occurred in the case or control within an exposure period, respectively. N indicates no, and Y indicates yes, namely a sepsis event occurred.

Subgroup analyses were based on sex and the comorbid condition for CKD. No substantial difference was observed in dialysis risk between male and female patients in the subgroup analysis (Figure 3 and Figure 4). The effect of sepsis on the risk of acute dialysis in patients with and without previous CKD was still observed (Figure 5 and Figure 6). The effect of sepsis on the risk of acute dialysis in patients without CKD was higher than in patients with CKD.

In analyses using no overlapping time intervals for exposure periods (Supplementary Figures S1 and S2), the increased risk of requiring acute temporary dialysis after sepsis was the greatest (15.8-fold) in the first 7 days following sepsis. This increased risk decreased markedly to five-fold from 8 to 14 days after sepsis but remained high (4.5-fold) for as long as 22–28 days after sepsis. The pattern for the first chronic dialysis requirement was somewhat different, with a 6.7-fold increased risk in the first 7 days after sepsis and a marked decrease to a two-fold increased risk from 8 to 14 days after sepsis, as well as in the 22–28 days interval.

## 4. Discussion

This case-crossover study demonstrated several key findings. First, patients with kidney injury displayed an increased dialysis risk after sepsis of nearly 15.8- and 6.7-fold when requiring acute temporary and chronic dialysis within one week. The risk remained high for up to four weeks after sepsis but gradually decreased as the time interval extended. Second, the risk was consistent after stratification based on gender. Third, in analyses using non-overlapping time intervals, the risk remained the highest during the time window closest to the index date.

The incidence of AKI requiring dialysis in patients with severe sepsis has increased in recent years, which may be attributed to early dialysis initiation associated with better survival outcome in severe AKI cases [28]. The baseline characteristics in patients with kidney injury may vary; therefore, the novelty of our study is the use of a case-crossover design to minimize within-person confounding bias.

Several pathophysiological mechanisms specific to sepsis-induced AKI have been proposed, including ischemic/hypoxic, nephrotoxic, and inflammatory insults, but no single pathway can explain all the features of septic AKI [29,30,31]. Ischemic injuries are commonly considered the leading cause of AKI, but sepsis-induced AKI can occur even without renal hypoperfusion and clinical signs of hemodynamic instability [32] and with normal or increased global renal blood flow [33].

Chronic medical conditions, such as diabetes mellitus, hyperlipidemia, and hypertension increase the risk of both sepsis and renal failure. The CCI is a widely used clinical scoring system, which predicts prognosis based on the patient’s comorbidities. In a retrospective study of 786 critically ill patients admitted with AKI or developing AKI during their hospital stay, a CCI score of >6 independently predicted poor renal outcomes [34]. Additionally, drug-induced nephrotoxicity varies from a relatively mild form of acute tubulointerstitial nephritis (ATIN), several types of glomerulonephritis, crystal nephropathy, and osmotic nephrosis to acute tubular necrosis represented mostly by severe renal impairment with the need for renal replacement therapy. The incidence of medication-associated nephrotoxicity accounts for approximately 18–27% of all AKI patients in US hospitals—the main causative drugs are NSAIDs, aminoglycosides, amphotericin B, and calcineurin inhibitors [35,36]. Therefore, close monitoring of renal function is necessary when administering antibiotics to septic patients; adequate intravenous hydrations, avoidance of co-administering nephrotoxic agents, and alternative antibiotic regimens should be considered in patients with CKD and sepsis.

Several studies have demonstrated that preexisting CKD is a key factor in the development of AKI and progression of end-stage kidney disease (ESKD) [37,38]. A large community-based cohort of patients with CKD found that patients with baseline-estimated glomerular filtration rates of 30–44 mL/min per 1.73 m^2^ had a 42% risk of developing ESKD, while those with rates 15–29 mL/min per 1.73 m^2^ had a 63% risk [39]. Even a single acute insult to already-compromised kidneys may be devastating. The mechanism linking CKD progression and AKI are not yet fully understood, but recent studies suggest a relationship between persistent kidney injury induced by sepsis-associated AKI and systemic inflammation [39,40].

To the best of our knowledge, this is the first study using case-crossover design to investigate the risk of kidney injury requiring acute temporary or chronic dialysis after sepsis. Our study provides strong evidence that sepsis significantly increases the risk of kidney injury requiring dialysis, both acute temporary and chronic. The risk remains high for up to 4 weeks after sepsis but gradually decreases as the time interval extends. Healthcare providers should be vigilant in monitoring renal function in septic patients, especially those with preexisting CKD, and take necessary precautions to minimize the risk of AKI and its progression to ESKD. Moreover, the early identification of patients at risk of AKI and the discovery of biomarkers associated with AKI that can aid in the early diagnosis is an area of intensive investigation. Timely and appropriate medical intervention, ongoing monitoring, and comprehensive support systems are crucial for managing the implications and improving outcomes for individuals affected by sepsis-related kidney damage and reduce the disease burden of CKD requiring dialysis. Further research is needed to better understand the underlying mechanisms and design strategies to prevent and manage sepsis-induced AKI.

The present study has several strengths. First, this was a national population-based study, which included a sufficiently large sample size, thus increasing the generalizability of the study. Acute temporary or chronic dialysis can be identified correctly by the procedure codes used in the claim database. Second, we used a case-crossover design based on within-individual comparisons. This self-matching of cases reduces control-selection bias, removes the effect of genetic factors, and minimizes confounding bias through adjusting time-invariant confounding factors. Finally, evaluating the different length of exposure periods as well as nonoverlapping time windows provided more insights into the risk of dialysis after sepsis. We observed that the risk of dialysis remained the highest at the time closest to sepsis.

This study also has several limitations. First, sepsis diagnosis was identified on the basis of ICD-9-CM codes, and we have no detailed information to define the severity of sepsis. Therefore, we are unable to estimate a severity-related relation between sepsis and the risk of dialysis. Second, the NHI Research Database in Taiwan does not provide detailed information on health-related factors, such as body mass index, history of smoking, and dietary habits, all of which may be confounders. To combat this, we used the case-crossover design to minimize the confounding bias; that is, the status for these confounders may not change in a short time period (six months). Third, due to the case-crossover design, all patients who underwent dialysis were six months older than those in the control group—age is a major risk factor for CKD and dialysis initiation in these patients. However, the age effect for six months might be very small. Owing to these limitations, a large prospective study in future may provide helpful information to clinicians.

## 5. Conclusions

This study demonstrated nearly 15.8- and 6.7-fold increases in the risks of acute temporary and chronic dialysis within 1 week after sepsis exposure, and patients remain at risk of acute temporary or chronic dialysis as long as 4 weeks after sepsis exposure. Patients with sepsis and AKI may require acute temporary dialysis, but some patients may be unable to recover and thus require long-term chronic dialysis. The medical cost of patients with CKD requiring chronic dialysis is enormous and has become a serious public health concern. This study identified a unique group of patients at increased risk of dialysis. To reduce the disease burden of CKD requiring dialysis, taking effective precautions in those with high risk is important. Further studies are needed to confirm these relationships in other populations to determine the exact mechanisms of the increased risk of kidney failure requiring dialysis after sepsis and to develop effective strategies to reduce this risk.

## Figures and Tables

**Table 1:** Characteristics of patients with acute temporary dialysis and chronic dialysis.

	Acute Temporary Dialysis		Chronic Dialysis	
	Case	Self-Control	Case	Self-Control
N	147,201		75,031	
Age at first dialysis, mean ± SD, y	68.2 ± 15.7		63.7 ± 14.5	
Age at first dialysis, N (%)				
20–29 y	2482 (1.7)		1287 (1.7)	
30–39 y	5849 (4.0)		3450 (4.6)	
40–49 y	11,563 (7.9)		7651 (10.2)	
50–59 y	21,178 (14.4)		15,796 (21.1)	
60–69 y	26,852 (18.2)		18,037 (24.0)	
70–79 y	38,645 (26.3)		18,238 (24.3)	
80–89 y	34,529 (23.5)		9646 (12.9)	
≥90 y	6103 (4.2)		926 (1.2)	
Male, N (%)	87,069 (59.2)		40,081 (53.4)	
Year of first dialysis, N (%)				
2006	12,659 (8.6)		6290 (8.4)	
2007	13,745 (9.3)		6690 (8.9)	
2008	14,224 (9.7)		7064 (9.4)	
2009	14,554 (9.9)		7329 (9.8)	
2010	15,237 (10.4)		7706 (10.3)	
2011	15,525 (10.6)		7180 (9.6)	
2012	15,357 (10.4)		7754 (10.3)	
2013	15,173 (10.3)		8092 (10.8)	
2014	15,217 (10.3)		8264 (11.0)	
2015	15,510 (10.5)		8662 (11.5)	
Comorbid conditions, N (%)				
Diabetes mellitus	68,499 (46.5)	60,588 (41.2)	43,552 (58.1)	40,567 (54.1)
Hypertension	94,917 (64.5)	82,203 (55.8)	65,448 (87.2)	53,629 (71.5)
Hyperlipidemia	26,103 (17.7)	25,233 (17.1)	20,108 (26.8)	19,602 (26.1)
Charlson comorbidity index, N (%)				
≤3	68,342 (46.4)	86,738 (58.9)	28,217 (37.6)	33,701 (44.9)
4–5	38,370 (26.1)	33,797 (23.0)	24,674 (32.9)	22,373 (29.8)
>5	40,489 (27.5)	26,666 (18.1)	22,140 (29.5)	18,957 (25.3)
Charlson comorbidity index, mean ± SD	4.1 ± 2.7	2.5 ± 2.4	4.4 ± 2.1	3.1 ± 2.2
Treatment with NSAIDs within 1 year prior to first dialysis, N (%)	50,017 (34.0)	48,963 (33.3)	21,030 (28.0)	21,652 (28.9)

**Table 2:** Characteristics of patients with acute temporary dialysis stratified by chronic kidney disease.

Acute Temporary Dialysis	Non-CKD		CKD	
	Case	Self-Control	Case	Self-Control
N	79,964		67,237	
Age at first dialysis, mean ± SD, y	65.8 ± 16.9		71.0 ± 13.6	
Age at first dialysis, N (%)				
20–29 y	2000 (2.5)		482 (0.7)	
30–39 y	4646 (5.8)		1203 (1.8)	
40–49 y	8281 (10.4)		3282 (4.9)	
50–59 y	12,771 (16.0)		8407 (12.5)	
60–69 y	13,793 (17.3)		13,059 (19.4)	
70–79 y	18,439 (23.1)		20,206 (30.1)	
80–89 y	16,787 (21.0)		17,742 (26.4)	
≥90 y	3247 (4.1)		2856 (4.3)	
Male, N (%)	49,805 (62.3)		37,264 (55.4)	
Year of first dialysis, N (%)				
2006	6852 (8.6)		5807 (8.6)	
2007	7464 (9.3)		6281 (9.3)	
2008	7868 (9.8)		6356 (9.5)	
2009	8028 (10.0)		6526 (9.7)	
2010	8319 (10.4)		6918 (10.3)	
2011	8349 (10.4)		7176 (10.7)	
2012	8225 (10.3)		7132 (10.6)	
2013	8071 (10.1)		7102 (10.6)	
2014	8314 (10.4)		6903 (10.3)	
2015	8474 (10.6)		7036 (10.5)	
Comorbid conditions, N (%)				
Diabetes mellitus	27,315 (34.2)	22,646 (28.3)	41,184 (61.3)	37,942 (56.4)
Hypertension	39,703 (49.7)	34,068 (42.6)	55,214 (82.1)	48,135 (71.6)
Hyperlipidemia	10,568 (13.2)	9927 (12.4)	15,535 (23.1)	15,306 (22.8)
Charlson comorbidity index, N (%)				
≤3	50,473 (63.1)	60,934 (76.2)	17,869 (26.6)	25,804 (38.4)
4–5	15,932 (19.9)	12,143 (15.2)	22,438 (33.4)	21,654 (32.2)
>5	13,559 (17.0)	6887 (8.6)	26,930 (40.1)	19,779 (29.4)
Charlson comorbidity index, mean ± SD	3.2 ± 2.7	1.7 ± 2.1	5.1 ± 2.4	3.4 ± 2.3
Treatment with NSAIDs within 1 year prior to first dialysis, N (%)	24,305 (30.4)	23,228 (29.1)	25,712 (38.2)	25,735 (38.3)

CKD: chronic kidney disease; SD: standard deviation.

## Data Availability

The data that support the findings of this study are available from the National Health Insurance Research Database in Taiwan. Restrictions may apply to their availability because the data were acquired under the license for the current study. Data cannot be shared by the authors or made public for ethical/privacy reasons.

## Supplementary Materials

The following supporting information can be downloaded at: https://www.mdpi.com/article/10.3390/jcm12154950/s1, Figure S1: Odds ratios of acute temporary dialysis at 1st, 2nd, 3rd, and 4th weeks before the index date when control was selected from six months before first dialysis; Figure S2: Odds ratios of chronic dialysis at 1st, 2nd, 3rd, and 4th weeks before the index date when control was selected from six months before first dialysis.
