# Supplementary material for: A National Case-Crossover Study on the Risk of Kidney Injury Requiring Dialysis after Sepsis
Source: J Clin Med. 2023 Jul 27;12(15):4950. doi: 10.3390/jcm12154950 (PMC10419702; doi:10.3390/jcm12154950)
Supplement: Supplementary file 1 [file jcm-12-04950-s001.zip › jcm-2372862-supplementary.pdf]

SUPPLEMENTAL MATERIAL

Supplemental Figures Legends

Supplement Figure S1. Odds ratios of acute-temporary dialysis at 1st, 2nd, 3rd, and 4th week after sepsis when control was selected from six months before first dialysis.

Supplement Figure S2. Odds ratios of chronic dialysis at 1st, 2nd, 3rd, and 4th week after sepsis when control was selected from six months before first dialysis.

Supplement Figure S1 Odds ratios of acute-temporary dialysis at 1st, 2nd, 3rd, and 4th week after sepsis when control was selected from six months before first dialysis.

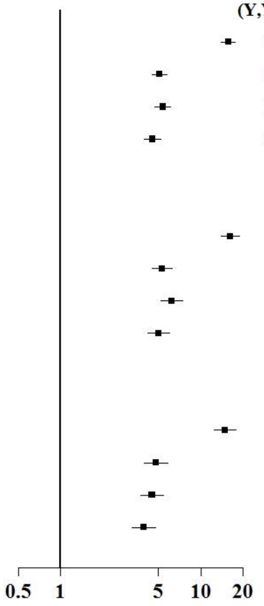

All patients (N=147201)	Concordant Pairs (Y,Y) or (N,N)	Discordant Pairs (Y,N)	Discordant Pairs (N,Y)	Odds ratio(95% C.I.)
Exposure period				
1- 7 days	142002	4889	310	15.77 (14.06-17.69)
8-14 days	145345	1551	305	5.09 (4.50-5.75)
15-21 days	145411	1509	281	5.37 (4.73-6.10)
22-28 days	145697	1232	272	4.53 (3.97-5.16)
**Men (N=87069)**				
Exposure period				
1- 7 days	83914	2973	182	16.34 (14.06-18.97)
8-14 days	85960	933	176	5.30 (4.51-6.23)
15-21 days	86033	892	144	6.19 (5.19-7.39)
22-28 days	86194	730	145	5.03 (4.21-6.02)
**Women (N=60132)**				
Exposure period				
1- 7 days	58088	1916	128	14.97 (12.52-17.90)
8-14 days	59385	618	129	4.79 (3.96-5.79)
15-21 days	59378	617	137	4.50 (3.74-5.42)
22-28 days	59503	502	127	3.95 (3.25-4.80)

Supplement Figure S2 Odds ratios of chronic dialysis at 1st, 2nd, 3rd, and 4th week after sepsis when control was selected from six months before first dialysis.

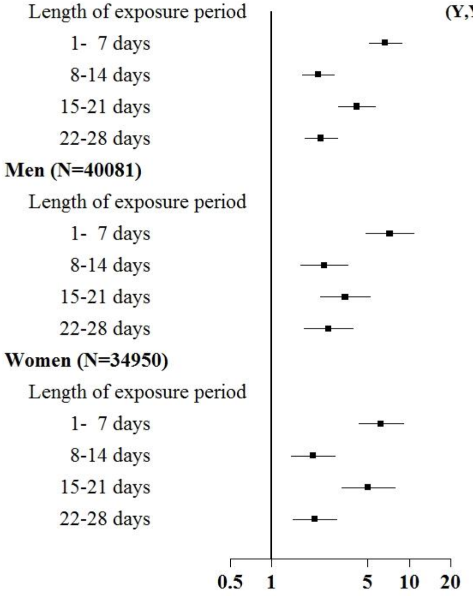

	Concordant Pairs (Y,Y) or (N,N)	Discordant Pairs (Y,N)	Discordant Pairs (N,Y)	Odds ratio(95% C.I.)
**All patients (N=75031)**				
Length of exposure period				
1- 7 days	74577	395	59	6.69 (5.09-8.80)
8-14 days	74783	170	78	2.18 (1.67-2.85)
15-21 days	74768	212	51	4.16 (3.06-5.64)
22-28 days	74788	169	74	2.28 (1.74-3.00)
**Men (N=40081)**				
Length of exposure period				
1- 7 days	39859	195	27	7.22 (4.83-10.80)
8-14 days	39962	84	35	2.40 (1.62-3.56)
15-21 days	39957	96	28	3.43 (2.25-5.22)
22-28 days	39966	83	32	2.59 (1.73-3.90)
**Women (N=34950)**				
Length of exposure period				
1- 7 days	34718	200	32	6.25 (4.30-9.08)
8-14 days	34821	86	43	2.00 (1.39-2.88)
15-21 days	34811	116	23	5.04 (3.22-7.89)
22-28 days	34822	86	42	2.05 (1.42-2.96)
